# Transcriptome Analysis and Screening for Potential Target Genes for RNAi-Mediated Pest Control of the Beet Armyworm, *Spodoptera exigua*

**DOI:** 10.1371/journal.pone.0065931

**Published:** 2013-06-18

**Authors:** Hang Li, Weihua Jiang, Zan Zhang, Yanru Xing, Fei Li

**Affiliations:** 1 Department of Entomology, College of Plant Protection, Nanjing Agricultural University, Nanjing, China; 2 Key Laboratory of Integrated Management of Crop Diseases and Pests, Ministry of Education, Nanjing Agricultural University, Nanjing, China; Swedish University of Agricultural Sciences, Sweden

## Abstract

The beet armyworm, *Spodoptera exigua* (Hübner), is a serious pest worldwide that causes significant losses in crops. Unfortunately, genetic resources for the beet armyworm is extremely scarce. To improve these resources we sequenced the transcriptome of *S. exigua* representing all stages including eggs, 1^st^ to 5^th^ instar larvae, pupae, male and female adults using the Illumina Solexa platform. We assembled the transcriptome with Trinity that yielded 31,414 contigs. Of these contigs, 18,592 were annotated as protein coding genes by Blast searches against the NCBI nr database. It has been shown that knockdown of important insect genes by dsRNAs or siRNAs is a feasible mechanism to control insect pests. The first key step towards developing an efficient RNAi-mediated pest control technique is to find suitable target genes. To screen for effective target genes in the beet armyworm, we selected nine candidate genes. The sequences of these genes were amplified using the RACE strategy. Then, siRNAs were designed and chemically synthesized. We injected 2 µl siRNA (2 µg/µl) into the 4^th^ instar larvae to knock down the respective target genes. The mRNA abundance of target genes decreased to different levels (∼20–94.3%) after injection of siRNAs. Knockdown of eight genes including chitinase7, PGCP, chitinase1, ATPase, tubulin1, arf2, tubulin2 and arf1 caused a significantly high level of mortality compared to the negative control (P<0.05). About 80% of the surviving insects in the siRNA-treated group of five genes (PGCP, chitinase1, tubulin1, tubulin2 and helicase) showed retarded development. In chitinase1-siRNA and chitinase7-siRNA administered groups, 12.5% survivors exhibited “half-ecdysis”. In arf1-siRNA and arf2-siRNA groups, the body color of 15% became black 48 h after injections. In summary, the transcriptome could be a valuable genetic resource for identification of genes in *S. exigua* and this study provided putative targets for RNAi pest control.

## Introduction

The beet armyworm, *Spodoptera exigua*, is one of the most serious agricultural pests of vegetable and flower crops in many agricultural areas all over the world [1]. In the 1950s, this pest destroyed young citrus trees and nursery stocks in California, leading to substantial economic losses in approximately 500 acres of citrus areas [2]. It is also a major pest of tomato in the Southeastern USA. The early infestations of this pest significantly correlated with yield loss in tomato [3]. The beet armyworm undergoes complete metamorphosis with a life cycle that consists of four different stages; eggs, five larval instars, pupae and adults. Larvae feed on both foliage and fruit of crops. As a leaf feeder, beet armyworm eats more cabbages than the diamondback moth, *Plutella xylostella* (Linnaeus), but is less damaging than the cabbage looper, *Trichoplusia ni* (Hübner) [4]. Control of this notorious pest is achieved by chemical pesticides. However, since overuse of pesticides leads to environmental and food safety problems it is highly desirable to develop alternate pest control strategies [5].

RNA interference (RNAi) is a powerful tool to knock down gene expression. When exogenous double-stranded RNAs (dsRNAs) are introduced into cells, they are processed into short interference RNAs (siRNAs) of 21–23 nt by the enzyme, Dicer. Then, the siRNA duplexes are incorporated into an RNA-induced silencing complex (RISC). Argonaute proteins, the catalytic components of RISC, use siRNA as a template to recognize and degrade the complementary messenger RNA (mRNA) [6], [7]. This RNAi technique has been successfully applied to study gene functions in many insects, including *Drosophila melanogaster* [8], *Tribolium castaneum* [9], *Nasonia vitripennis* [10], *Gryllus bimaculatus* [11], *Blattella germanica* [12], *Reticulitermes flavipes* [13], *Nilaparvata lugens* [14], [15] and *Epiphyas postvittana* [16]–[19]. There are two kinds of RNA delivery methods, oral intake or injection. Injection of siRNA or dsRNA is widely used in lab at a small-scale level, whereas oral intake is feasible to be used for controlling pest in field.

RNAi-mediated pest control is a new and promising technique because interference with important insect genes using RNAi can lead to death of pests [20], [21]. This breakthrough occurred in 2007, when RNAi-directed knock down of insect target genes in transgenic plants was shown to protect plants from coleopteran and lepidopteran pests, opening a new era for generation of insect-resistant crops [22], [23]. Environmental RNAi, which refers to sequence-specific gene silencing by environmentally encountered dsRNA is an important basis for RNAi-mediated pest control because dsRNA or siRNA used to control pests should be delivered environmentally [24]. Fortunately, it was reported that trehalase and chitin synthase gene A in the beet armyworm could be successfully knocked down by injection or ingestion of bacteria expressing dsRNA [18], [25], suggesting that use of RNAi to control this pest is feasible.

To develop RNAi-mediated pest control methods, it is critical to find suitable target genes. Target genes should not only have insecticidal effects on the target pests, but should also be safe to non-target organisms. Unfortunately, the genetic resources for beet armyworm are extremely scarce and therefore additional resources are required for effective screening of target genes. Since transcriptomes have been reported to be useful genetic resources for high-throughput screening of RNAi target genes [26], we sequenced the transcriptome of the beet armyworm and then selected nine candidate genes to study their potential application in RNAi mediated pest control.

## Results

### Analysis of *S. exigua* Transcriptome

To create a useful genetic resource for the beet armyworm, we sequenced its transcriptome using Illumina Solexa platform. To obtain as many different transcripts as possible, we pooled the total RNAs from different developmental stages, including eggs, larvae, pupae and adults. After filtering, ∼34 million high quality reads remained. The average length of the reads was 76 bp. These reads were assembled into 31,414 contigs using the software Trinity with default parameters (Table 1). The contig N50 was 542 bp with lengths ranging from 202 to 9633 bp. We annotated 18,592 contigs to known proteins by performing Blastx searches against the NCBI nr database (E-value< = 10^−5^). The RNA-Seq data were submitted to the NCBI SRA database with the accession number SRA056289. This Transcriptome Shotgun Assembly project has been deposited at DDBJ/EMBL/GenBank under the accession GAFU00000000. The version described in this paper is the first version. This transcriptome provides a useful resource with a large number of genes from the beet armyworm.

**Table 1:** Summary of beet armyworm transcriptome data sequenced by Illumina Solexa platform.

Total number of reads	34,809,334
Total base pairs (bp)	2,645,509,384
Total number of contigs	31,414
Mean length of contigs (bp)	542
Sequences with E-value < = 10^−5^	18592
GC percentage	45.7%

**Figure 1:**
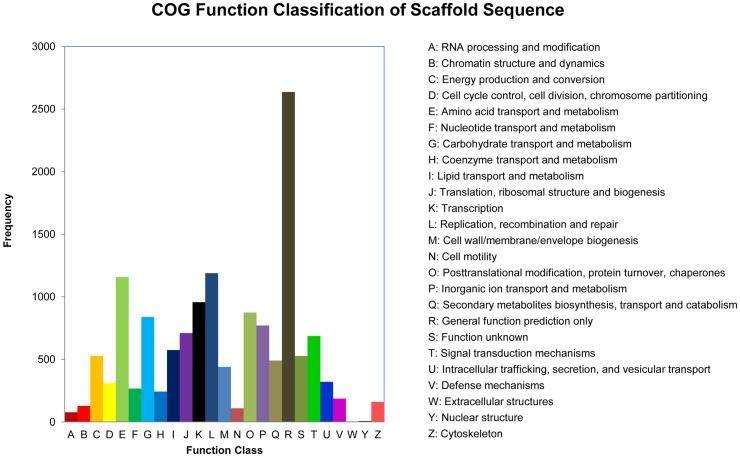
COG functional classification of the beet armyworm transcriptome. Orthologous analysis of 18,592 Trinity contigs was performed using the Blastall software against Cluster of Orthologous Groups (COG) database. The clusters “general function prediction only” and “Replication, recombination and repair” were abundant in the beet armyworm transcriptome data.

**Figure 2:**
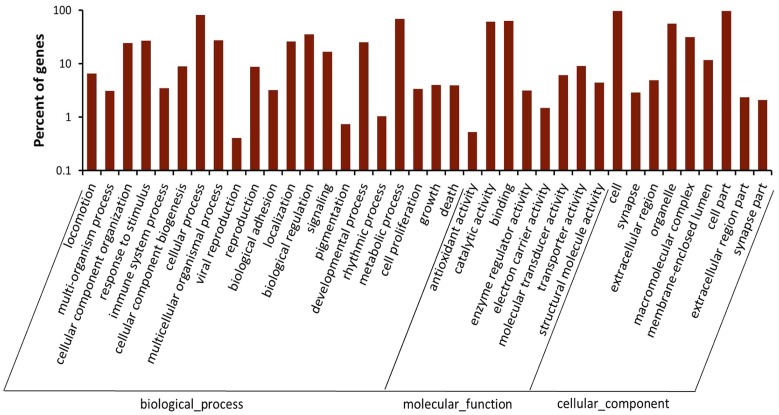
Gene ontology classification of the beet armyworm transcriptome. Blast2GO was used to analyze the 18,592 contigs identified by Blastx as having significant homology to genes in the NCBI nr database. “Cellular process” and “metabolic process” in biological process, “catalytic activity” and “binding” in molecular function, “cell” and “cell part” in cellular component were the most abundant.

We first removed redundant sequences in the transcriptome and then annotated the sequences based on COG and KEGG databases. Orthologous analysis using COG database demonstrated that the largest group “general function prediction only” contained 2,636 sequences, accounting for 18.6%. The second group was “Replication, recombination and repair”, which had 1,188 sequences (8.4%) (Fig. 1). GO annotation indicated that 10,259 sequences were categorized into 38 functional groups (Fig. 2). The dominant categories were “binding”, “cellular process” and “cell”.

### Amplifying the Transcripts of the Candidate Genes

Based on sequence similarities with known target genes in the corn rootworms and the importance of genes in cellular process, we chose nine candidate genes to study their potential application in RNAi-mediated pest control. These genes were ADP-ribosylation factor 1 (arf1), ADP-ribosylation factor 2 (arf2), tubulin1, tubulin2, chitinase1, chitinase7, plasma glutamate carboxypeptidase (PGCP), helicase and ATPase. Among these genes, PGCP, which plays a key role in the metabolism of secreted peptides [27] was chosen for the first time as a candidate in this study, whereas others have been studied in the corn rootworms [28].

We used Rapid Amplification of cDNA Ends (RACE) to amplify the transcripts of the selected candidate genes. We successfully amplified the 5′-UTR sequences of six genes (arf1, arf2, tubulin2, chitinase7, PGCP, helicase), but only obtained the complete 3′-UTR sequence of arf2. We assembled the 5′UTR, fragments from transcriptome and 3′UTR into a single transcript. Only the full-length transcript of arf2 gene was successfully obtained. Five genes (arf1, arf2, tubulin2, chitinase7, helicase) had an intact CDS (Table 4). These sequences shared high similarities with their orthologs in other insects (Table S2). All sequences were submitted to the GenBank database.

### Knock Down of Candidate Genes by siRNAs Injection

**Figure 3:**
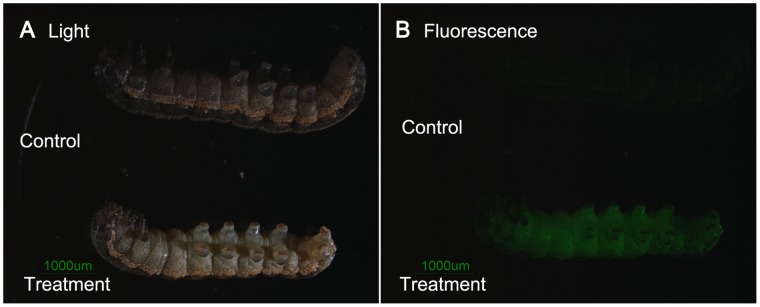
Observation of siRNA spread in the larvae after injection by fluorescent microscopy. Larvae injected with fluorescence labeled chitinase1-siRNA were observed under light (A) and fluorescent microscopy (B), which shows the immediate spread of siRNA to the whole body. The photos were taken five minutes after injection. The controls were un-injected larvae.

The siRNAs were injected into the 4^th^ instar larvae at the lateral region of the intersegment membrane between the first and second abdominal segments. To examine whether the siRNA successfully spread to the whole body, we used 2 µl fluorescence-labeled chitinase1 siRNA (2 µg/µl) for observation. Five minutes after injections, observations under a fluorescence microscope showed that siRNAs spread to the whole body (Fig. 3). For RNAi experiments, we injected 2 µl (2 µg/µl) non-labeled siRNA into the larvae. After injecting siRNAs, we selected three siRNA-treated insects at 24, 48, 72, 96 and 120 h (5 time points for 9 genes), respectively. These insects were frozen in liquid nitrogen until RNA purification to examine the RNAi effect. Unfortunately, RNA extraction from larvae treated with PGCP (24 h), chitinase1 (96 h), and tubulin2 (120 h) were not successful. Therefore, we did not obtain reliable data for these genes at these time points. However, in total 42 time points were obtained for nine target genes.

**Figure 4:**
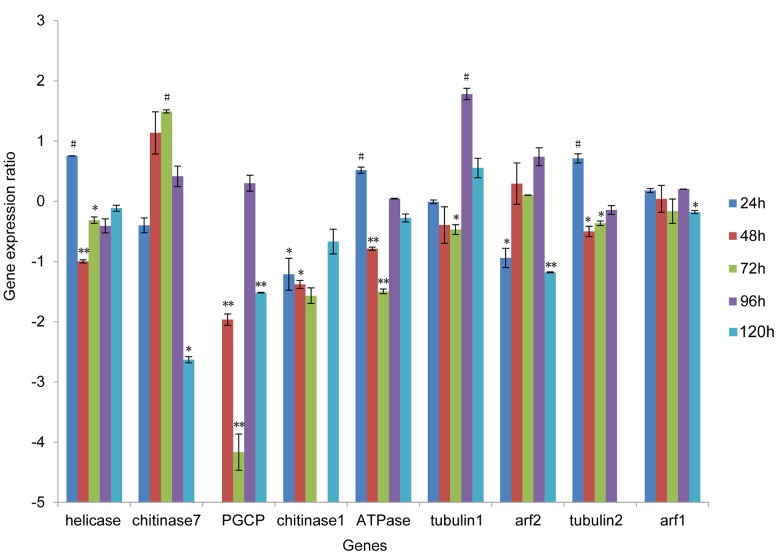
Relative mRNA abundance of nine target genes after injecting siRNAs. The mRNA levels of target genes were monitored by qRT-PCR at 24, 48, 72, 96 and 120 h after injection. Three insects were collected for each time point. The data for PGCP at 24 h, chitinase 1 at 96 h, and tubulin2 at 120 h are absent because of the unsuccessful RNA purification. Larvae injected with siRNA with a random sequence were used as negative controls. Two housekeeping genes, G3PDH and E2F, were used as multiple internal controls. The qRT-PCR data were analyzed using the delta-delta Ct method. The mRNA abundance of target genes in the negative control was used as the calibrator sample. The mRNA levels of target genes in the siRNA-treated group were relative to the negative control group at the same time point. Error bars indicate standard errors. Statistical significance of differences were analyzed with student T-test (decreasing significance,* P<0.05, **P<0.01; increasing significance, # P<0.05 ).

Quantitative RT-PCRs (qRT-PCR) were carried out to detect the mRNA levels of each gene in the injected and control groups. Based on statistical analysis, 16 of the 42 time points showed significant knockdown of mRNA levels (P<0.05), suggesting that injection of siRNA could induce gene silencing in *S. exigua*. Statistically significant reductions in mRNA abundance were observed for each target gene at least for one time point. However, the decreasing levels of mRNAs ranged from ∼20 to 95%, suggesting that RNAi efficiencies were not similar in the different genes. We reasoned that it might be ascribed to the differences in siRNA efficiency. At 72 h after injecting siRNAs, the expression of PGCP decreased to 5.69% of the control level (decreasing by 4.17 fold), which was the most efficient interference. The mRNA abundances of four genes (chitinase7, PGCP, arf1 and arf2) decreased to a statistically significant low level at 120 h after treatment (Fig. 4). The duration and degree of knockdown was variable for the nine genes tested. While several genes showed significantly lower transcript levels 48–72 h post injection (helicase, PGCP, ATPase, tubulin 2), for other genes the knockdown was not apparent until 120 h post injection (chitinase 7 and arf1).

We also observed the statistically significant increase in mRNA abundance at five time points (indicated by # in Figure 4), including at 24 h for helicase, 72 h for chintinase7, 24 h for ATPase, 96 h for tubulin1 and 24 h for tubulin2. At 96 h, the abundance of tubulin1 was 3.44 fold higher than the control level and was the highest increase among the target genes. At 72 h after injection, the expression of chitinase7 increased to 2.81 fold. In other RNAi experiments, we also found an increase in transcript abundance of target genes, which requires further investigation.

### Mortality

**Figure 5:**
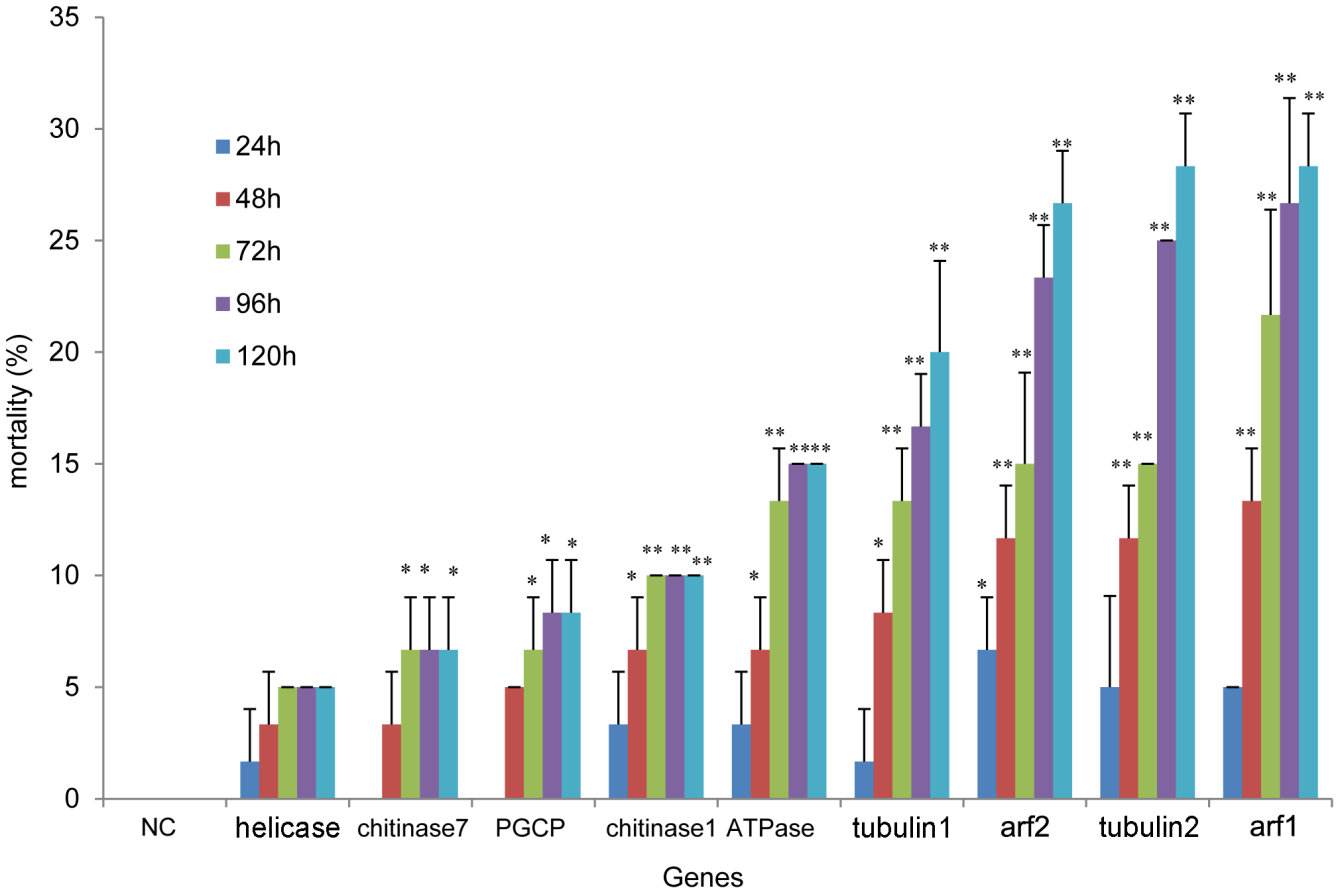
The mortality in RNAi-treated groups at different times after injection. The mortalities were calculated at 24, 48, 72, 96 and 120 h after injecting siRNAs. The group injected with siRNA with a random sequence was used as the negative control (NC). We did not observe mortality in the NC group. No mortality was observed at 24 h post injection in the chitinase 7 and PGCP siRNA group. Knockdown of eight genes led to significantly high mortality at 72, 96 and 120 h after siRNA injection. All data were analyzed with student T-test (* P<0.05, **P<0.01).

Mortality in RNAi-treated groups were calculated for nine target genes at 24, 48, 72, 96 and 120 h after injection for each gene, yielding 45 time points in total. Individual animals that did not move after touching with a small Chinese writing brush were counted as dead. For several of the target genes, the mortality of RNAi-treated groups continually increased with time. Knockdown of eight genes (chitinase7, PGCP, chitinase1, ATPase, tubulin1, arf2, tubulin2 and arf1) caused significantly high mortality at 72, 96 and 120 h after siRNA injection (P<0.05). The highest mortality (28.3%) appeared in the group injected with tubulin2-siRNA and arf1-siRNA (Fig. 5).

### Retarded Development and Abnormal Phenotypes

**Figure 6:**
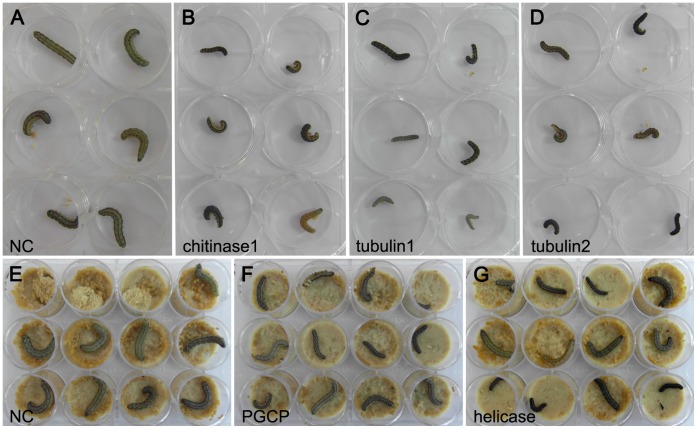
Retarded development in RNAi-treated groups. About 80% of surviving larvae in each siRNA-treated group showed retarded development. The group injected with siRNA with a random sequence was used as the negative control (NC). All photos were taken 48 h after injecting siRNAs. All the treatments are in same condition. To take good photos, we moved the insects to the lid of Petri dishes. However, in some cases, we took the picture directly with diet as the background.

**Figure 7:**
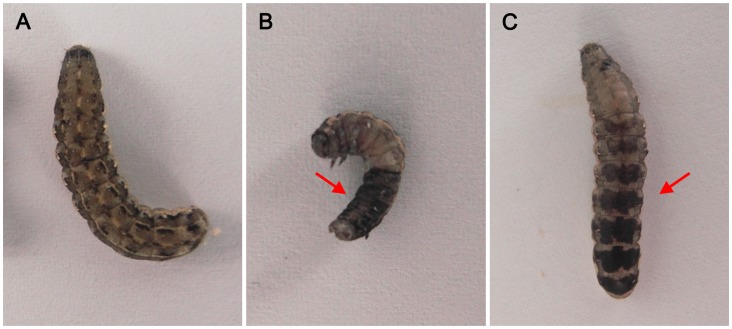
Developmental defects in siRNA-treated individuals. (A) Larvae in the negative control group injected with siRNAs with a random sequence. (B) The “half-ecdysis” in chitinase1-siRNA and chitinase7-siRNA groups. The photo was taken 24 h after siRNA injection. Red arrow indicates the part of larval body still in the old cuticle. This larva could not complete ecdysis and eventually died. (C) The “black body” in arf1-siRNA and arf2-siRNA groups. The photo was taken 72 h after siRNA injection. Red arrow indicates black color in the body.

Larval development in RNAi-treated groups was also observed. For each treatment, about 80% of surviving animals in the chitinase1-siRNA, tubulin1-siRNA, tubulin2-siRNA, PGCP-siRNA and helicase-siRNA groups showed retarded development (Fig. 6). RNAi of helicase gene did not lead to high mortality but the survivors showed apparent developmental defects. About 12.5% individuals in chitinase1-siRNA and chitinase7-siRNA groups exhibited “half-ecdysis” during the molting process, and approximately 70% of these individuals died. The body color of ∼15% insects in arf1-siRNA and arf2-siRNA groups became black at 48 h after treatment (Fig. 7) and then died within 2 days. We did not observe the “black color” phenotype and death in the negative control group, so it was unlikely that “black color” was caused by infection or injury. Some siRNA-treated individuals did not show any abnormal phenotypes because of siRNA degradation or individual difference in RNAi efficiency.

## Discussion

Although the beet armyworm is a very serious insect pest, its genome sequence is not available which makes the genetic resource for this pest scarce [29]. Here, we sequenced the transcriptome of the beet armyworm. The purposes for sequencing the transcriptome are not only to identify target genes for RNAi but also provide a valuable genetic resource. Pascual et al., also sequenced the transcriptome of beet armyworm using the Roche 454 FLX and Sanger methods. Their samples included larval tissues after immune activation by microbes [30]. Comparative analysis of the two transcriptome data indicated that Roche 454 FLX platform provided longer contigs, whereas Illumina Solexa platforms yielded shorter reads and yet identified low abundance transcripts because of deep coverage.

RNAi-mediated pest control is a hot topic in crop protection and has been investigated in various insect pests. In termites, feeding high-dose dsRNAs of two termite genes led to increase in mortality and fitness cost [13]. In the mosquito *Anopheles gambiae*, feeding larvae with chitosan/dsRNA nanoparticles repressed expression of two chitin synthase genes, AgCHS1 and AgCHS2, suggesting that nanoparticle-based RNAi technology can be used in pest control [31]. Feeding-based RNAi of a trehalose phosphate synthase gene was also performed in the brown planthopper, suggesting that this gene may also be a potential target gene in insect pest control [32]. Cotton bollworm larvae feeding on dsCYP6AE14-expressing cotton showed drastically retarded growth [33]. In the whitefly *Bemisia tabaci*, ds/siRNAs of five different genes (actin, ADP/ATP translocase, alpha-tubulin, ribosomal protein L9 (RPL9) and V-ATPase A subunit) via feeding caused 29–97% mortality [34]. A seven transmembrane-domain odorant receptor (OR) also proved to be a potential target for RNAi-based pest management of *Phyllotreta striolata* [35]. In the Colorado potato beetle, feeding dsRNAs successfully triggered silencing of all five target genes, leading to significant mortality [36].

In the beet armyworm, trehalase and chitin synthase gene A were reported to be potential targets for RNAi mediated pest control [18], [25]. To provide more candidate targets, we screened nine important genes in the beet armyworm and found that knockdown of tubulin1, arf2, tubulin2 and arf1 led to >20% mortality. Though the mortalities were not as high as expected, we still speculate that these genes could be potential target genes for further consideration in field application. Considering the cost of siRNA synthesis, we injected only 4 µg siRNAs to each larva. However, transgenic plants and/or genetically modified microorganisms can deliver high amounts of siRNAs continuously into the larvae. If higher doses of dsRNA can be administered environmentally, the efficiency of RNAi may be increased.

In this study, we used the injection method to deliver siRNAs to the larvae. It should be noticed that injection of siRNA is not applicable in the field. Because siRNA can be easily degraded in the environment, transgenic crops or bacteria expressing siRNA/dsRNA are normally considered as practical techniques to deliver RNAi for pest control [37], [38]. By these two methods, siRNA/dsRNA are taken into the midgut of larvae, while during injection siRNA/dsRNA are delivered into the hemolymph. Therefore, the difference in the method could apparently affect RNAi efficiency. The potential target genes identified in this study using the injection method should be further confirmed before they are used for field applications.

Our work provides some new insights into the discovery of target genes for RNAi mediated pest control. It has been reported that knockdown of helicase and chintinase7 can cause high mortality in western corn rootworm [28]. However, this was not the case in the beet armyworm. Although helicase was successfully knocked down to more than 50%, we did not observe high mortality in the siRNA-treated groups. This result suggests that RNAi of orthologous genes may not result in the same phenotype even in closely related species. Therefore, even though searching orthologous genes of known RNAi targets is helpful, potential target genes must be validated.

Another interesting phenomenon is that RNAi efficiencies are not positively related to mortalities. The highest mortalities appeared in the arf1-siRNA and tubulin2-siRNA treated groups. However, RNAi efficiencies in these two groups were only 20–30%. In contrast, high RNAi efficiencies in chitinase7-siRNA (71.7%) and PGCP-siRNA (94.3%) groups did not lead to high mortalities (6.67% and 8.3%, respectively). We reasoned that this may be due to the redundancy and importance of genes. If an important gene has low redundancy (meaning that there are no other genes with similar functions), knocking down this gene (e.g. arf1 and arf2) would lead to death. Thus, an efficient RNAi target gene must have two features, importance and low functional redundancy.

We also noticed that the RNAi effects were not consistent for most target genes. In a total of 45 time points in nine genes (5 time points for 9 genes), 24 time points showed decrease in mRNA abundance, but only 16 time points showed significantly lower levels (P<0.05) compared to the controls. The inconsistency in RNAi effects was observed in many experiments, suggesting that RNAi efficiency is hard to be controlled. There were seven time points with increase in mRNA abundance after siRNA treatments. We found similar phenomena of increase in mRNA abundance of RNAi target genes in other experiments [26]. We repeated the experiments and obtained similar results. Further work is needed to understand this mechanism.

### Conclusion

Transcriptome of the beet armyworm was sequenced with Illumina Solexa platform that yielded 31,414 contigs. Nine important genes were selected to study their potential application in RNAi-mediated pest control. We used RACE strategy to amplify the gene transcripts, and produced one full-length transcript and five intact CDSs. The candidate genes were knocked down by chemically synthesized siRNA to different levels. Interference of eight genes caused high mortality with significant difference (P<0.05), suggesting that these genes may be considered as potential RNAi target genes for further study. The survived individuals in the five RNAi-treated groups showed apparent developmental defects, indicating that these genes have important functions in insect development.

## Materials and Methods

### Insects

Larvae of the beet armyworm, *S. exigua*, were maintained on an artificial diet in the laboratory at 25±2°C and 75±5% relative humidity on a photoperiod (Light: Dark = 14∶10). To prepare sufficient RNAs for sequencing transcriptome, enough samples should be prepared: fifty eggs, twenty larvae from the 1^st^ and 2^nd^ instar, five larvae from the 3^rd^ instar and one larva each from the 4^th^ to 5^th^ instar, one pupa, one male and one female adult. These samples were collected and immediately frozen in liquid nitrogen and maintained at −70°C until further use. We purified total RNA from each sample and mixed equal amounts of each total RNA. For RNAi experiments, 4^th^ instar larvae were used.

### RNA Isolation and cDNA Library Construction

Total RNA was extracted using the SV total RNA isolation system according to the manufacturers’ protocol (Promega, USA). The quality of RNA was examined using the Agilent 2100 Bioanalyzer (Agilent Technologies). Beads containing oligo (dT) were used to isolate poly (A) mRNA from pooled total RNA (a mixture of total RNA from eggs, larvae, pupae and adults at equal ratio). The cDNA synthesis was followed by Illumina mRNA sequencing sample preparation guide. Fragmentation buffer (200 mM Tris-Acetate, pH 8.1, 500 mM KOAc, 150 mM MgOA) was added to shear mRNAs into short fragments, which were used as templates to synthesize the first-strand cDNAs with random hexamer primers. The second-strand cDNA was then synthesized according to the manufacturer’s protocol (Takara, Japan). Short cDNA fragments were purified with the QiaQuick PCR extraction kit and resolved with elution buffer (10 mM Tris·HCl, pH 8.5) for adding poly (A). Finally, the short cDNA fragments were linked to adaptors for sequencing.

### Bioinformatics Analysis of RNA-Seq Data

The cDNA libraries were sequenced using Illumina HiSeq™ 2000. The size of the cDNA library was approximately 200 bp and both ends of all clones were sequenced. Image de-convolution and quality value calculations were performed using the Illumina GA pipeline1.3. The empty reads, low quality sequences (reads with unknown sequences ‘N’) and the adaptor sequences of high quality reads were removed. The reads obtained were randomly clipped into 21 bp K-mers for assembly using *de Bruijn* graph and Trinity (r2012-10-05) [39], [40]. The Trinity combined reads with a certain length of overlap to form contigs. Then the raw reads were mapped back to contigs. By using paired-end reads, contigs from the same transcript and the distances between these contigs can be determined. Then, Trinity assembled these contigs to obtain consensus sequences that contained the least Ns. The assembled sequences were used for annotation by Blastx searches against the NCBI nr database using an E-value cut-off of 10^−5^. We wrote a Perl script to compare multiple isoforms of contigs assembled by Trinity and kept the longest one for further analysis. Functional annotation with gene ontology terms (GO, http://www.geneontology.org) was analyzed by Blast2go software with default parameters [41]. The orthologous and pathway analysis were performed using the Blastall software against Cluster of Orthologous Groups (COG) and Kyoto Encyclopedia of Genes and Genomes (KEGG) databases [42].

### RACE

SMART RACE cDNA amplification kit (Clontech, USA) was used to obtain the full-length transcripts. Total RNA was extracted using Trizol reagent (Invitrogen, USA), and the cDNA were synthesized as recommended in the SMART RACE kit. Gene-specific primers (GSP) and Nested gene-specific primers (NGSP) were designed for 5′- and 3′-RACE using the software primer premier 5.0. The primer sequences are listed in Table 2. The first round PCRs were performed with the GSP primer and Universal Primer Mix (UPM). The 1,000 X diluted first PCR products were used as the templates in the nested PCRs with NGSP. The RACE products were separated on an agarose gel and purified using the Wizard® SV Gel and PCR Clean-Up System (Promega, USA). Purified cDNA was ligated into the pGEM-T Easy Vector (Promega, USA) and sequenced completely from both directions. All RACE results were confirmed by end-to-end PCRs.

**Table 2:** Primers used in the experiments.

	Primer	Sequence (5′- 3′)
RACE	tubulin2 -5′-GSP	TCCCTGGCTCGAGGTCGAGCAG
	tubulin2 -5′-NGSP	TGGGGTCGATGCCGTGCTTCTC
	PGCP- 5′-GSP	TGATTCCTCCTCGTGGTGTACTGACGCT
	PGCP-5′-NGSP	TTTAAGCGTACCCAATGGGGAACCTG
	arf1- 5′-GSP	TCGTCGTCTTCCCGGCACCGTCCAAG
	arf1 -5′-NGSP	CACCGTCCAAGCCGAGGATCAAAATC
	arf2- 5′-GSP	GGTTTTACCAGCAGCATCCAAACCAACC
	arf2 -5′-NGSP	CCAGCAGCATCCAAACCAACCATCAA
	arf2- 3′-GSP	TCCGCCTACAGGGAACAGTGCTCAGA
	arf2 -3′-NGSP	TGAAAATGCGTATTGTGACATTCCGGTTT
	helicase- 5′-GSP	CCCCTTGAGGACGTGGTATCGCTCGA
	helicase-5′-NGSP	CAGCATCTAACGCACGACAAGAACCG
	ATPase - 5′-GSP	CAAATTCGTCTCCCCATCCAACTCGG
	ATPase -5′-NGSP	CCAGCCTGATGACGTCTCCCACCTGA
qRT-PCR	chitinase7-F	AACTGACTGAAGGAGACGAGAAGG
	chitinase7-R	CGAAAGCCCAACCACCAATAGC
	chitinase1-F	TGCCATTCTACGGTCAATCATTCTC
	chitinase1-R	TTCATCGCCAGCCTCTCCAC
	tubulin1-F	GCTGACTGCGTAATGGTGCTG
	tubulin1-R	TAGAGACGAGGGTGTTTATCTGTGC
	tubulin2-F	GGGCACGCTCCTAATCTCCAAG
	tubulin2-R	CGTTGTAGGGCTCCACCACTG
	PGCP-F	GCAATGGATGATGGTGGAGGTATG
	PGCP-R	TGCTCTCACTGTCCTTCTTGGTC
	arf1-F	CGGAGGCGTTTTAACTGAAGAGG
	arf1-R	AATGGCGTTACAAGTGACTATGCTG
	arf2-F	GCAAGCAGTATTCGGAAGCGTAG
	arf2-R	TCAACAGCCCAAACTCACAAAGC
	helicase-F	GATGTAGTAAATGGTGCGGGAAAGG
	helicase –R	TAGGGATGGTTGCGACACTTACG
	ATPase-F	CCGCCGTGTCCGTCGTG
	ATPase-R	CGTTCGTGTCGTCGTTGTGG
	G3PDH-F	GTAGTTTCCCACCAGTTTGTCAG
	G3PDH-R	CACCTCAGACGCAATGTTAGC
	E2F-F	GCCCAATCTGTTTCCTCAAAAG
	E2F-R	GAACTTGCTCGCCGTAAGAC

GSP: gene specific primer. NGSP: nest gene specific primer.

### RNA Interference

We designed siRNAs based on the sequences confirmed by end-to-end PCRs. The sequences of siRNAs are listed in Table 3. One set of siRNA with a random sequence was used as the negative control (NC), which did not share any sequence similarity with the transcriptome of beet armyworm using BlastX search. The siRNAs were chemically synthesized by Shanghai GenePharma Co., Ltd (Shanghai, China). To observe the spread of siRNAs in the larval body, we labeled the chitinase1-siRNA with the fluorescent dye, FAM at the 5′end of the sense strand. The siRNAs were mixed to form double-stranded siRNA, purified by high-performance liquid chromatography, and dissolved in the diethylpyrocarbonate treated water (Milli-Q grade). Then, 2 µl siRNAs (2 µg/µl) were injected into the 4^th^ instar larvae using microsyringe at the lateral region of the intersegment membrane between the first and second segments of the abdomen. The siRNA-treated individuals were separately maintained in 12-cell plates. The NC-siRNA was used as the negative control and injected into the larvae under the same conditions. Each treatment had 48 individuals, and was randomly divided into two sub-groups (24 for each sub-group). One sub-group was used for mortality analysis and observation of developmental defects. In this group, insect development and behavior after siRNA treatment were observed every day. Another sub-group was used to estimate the mRNA abundance after RNAi. Three insects were randomly chosen at 24, 48, 72, 96 and 120 h after injection of siRNA for quantitative RT-PCR (qRT-PCR). All experiments were independently repeated in triplicate. The NC group was also included for each time point. Levene’s Test was used to estimate the homogeneity of variances. The results indicated that the data had equal variances. Student T-test was then used to compare the data of RNAi-treated group with the NC group at same time point for analysis of statistical significance (P<0.05) using the SPSS software.

**Table 3:** siRNA sequences used in RNAi experiments.

SiRNA	Sense (5′-3′)	Antisense (5′-3′)
arf2	GGUGGUAAGUGUACAAAUATT	UAUUUGUACACUUACCACCTT
arf1	CGUGGCCUCUUCAGUUAAATT	UUUAACUGAAGAGGCCACGTT
tubulin1	GCUGCUUCAACCAAAGAAUTT	AUUCUUUGGUUGAAGCAGCTT
tubulin2	GGUGCACUAUUUCCCUCAUTT	AUGAGGGAAAUAGUGCACCTT
chitinase1	GCCGGAAGAAAUAGAAGAATT	UUCUUCUAUUUCUUCCGGCTT
chitinase7	GCUGGAGAAUUCACUUUAATT	UUAAAGUGAAUUCUCCAGCTT
PGCP	GGAGCAUCGUUGCUUAAUATT	UAUUAAGCAACGAUGCUCCTT
helicase	GGGCCUGGUACAUUCUUAATT	UUAAGAAUGUACCAGGCCCTT
ATPase	GCCCGCAAAUUUAUUUAAATT	UUUAAAUAAAUUUGCGGGCTT
NC	UUCUCCGAACGUGUCACGUTT	ACGUGACACGUUCGGAGAATT

**Table 4:** Sequence information of nine candidate genes in *S. exigua* amplified by RACE strategy.

Gene	Assemble length(bp)	CDS (bp)	5′UTR (bp)	3′UTR (bp)	Number of amino acids	GenBank ID
arf2	1941*	549**	122 **	1270 **	182	JF915770
arf1	1293	543**	204 **	539	180	JQ653045
tubulin1	1541	1371**	45	125	456	JQ653042
tubulin2	1066	609	101 **	356	202	JQ653043
chitinase1	4751	4413	106	196	1470	JQ653040
chitinase7	3141	2964**	136**	41	987	JQ653039
PGCP	1348	1317	26 **	–	439	JQ653044
helicase	3293	3069 **	173 **	51	1022	JF915771
ATPase	3328	3279	48	–	1092	JQ653046

* Full length

** intact CDS/5′UTR/3′UTR.

### Quantitative RT-PCR

The mRNA abundances were determined by qRT-PCR using an ABI PRISM 7500 (Applied Biosystems, USA) with FastStart Universal SYBR Green Master (Roche, Germany). Total RNA was purified from three insects collected from siRNA-treated group and the NC group using the SV total RNA isolation system according to the manufacturers’ protocol (Promega, USA). The cDNA was synthesized using the PrimeScript RT Master Mix perfect Real time kit (Takara, Japan). For qRT-PCR reactions, 50 ng cDNA was used as the template in 30 µl reactions. The cycling protocol was: one cycle of 95°C for 10 min, 40 cycles of 95°C for 15 s and 60°C for 60 s. The qRT-PCR primers are listed in Table 2. Two housekeeping genes, G3PDH and E2F, were used as internal controls for normalization of the data. The primer efficiencies were determined by cDNA template dilution and reached the requirements (Table S1). We used the qBase software for qRT-PCR data analysis with default parameters, which uses an improved delta-delta-Ct method. This method takes into account multiple reference genes [43]. The fold changes were log transformed before statistical significance of differences analysis using Student T-test.

## Supporting Information

Table S1 **The primer efficiency of nine test genes and two reference genes.**
(DOC)

Table S2 **The e-value and similarities of nine test genes with their orthologous in other insects.**
(DOCX)
