# Supplementary material for: Transcriptome Analysis and Screening for Potential Target Genes for RNAi-Mediated Pest Control of the Beet Armyworm, Spodoptera exigua
Source: PLoS One. 2013 Jun 18;8(6):e65931. doi: 10.1371/journal.pone.0065931 (PMC3688801; doi:10.1371/journal.pone.0065931)
Supplement: Table S1 — The primer efficiency of nine test genes and two reference genes. (DOC) [file pone.0065931.s001.doc]

**Table S1 the primer efficiency of nine test genes and two reference genes. The primer efficiencies were determined by cDNA template dilution method.**

| **Gene** | **Primer Efficiency** |
| --- | --- |
| arf1 | 1.02 |
| arf2 | 0.97 |
| tubulin1 | 0.94 |
| tubulin2 | 0.98 |
| chitinase1 | 0.92 |
| chitinase7 | 0.97 |
| PGCP | 1.05 |
| helicase | 0.90 |
| ATPase | 1.02 |
| G3PDH | 1.05 |
| E2F | 0.96 |

The primer efficiencies were all within 10% of each other.
