# Supplementary material for: Transcriptome Analysis and Screening for Potential Target Genes for RNAi-Mediated Pest Control of the Beet Armyworm, Spodoptera exigua
Source: PLoS One. 2013 Jun 18;8(6):e65931. doi: 10.1371/journal.pone.0065931 (PMC3688801; doi:10.1371/journal.pone.0065931)
Supplement: Table S2 — The e-value and similarities of nine test genes with their orthologous in other insects. (DOCX) [file pone.0065931.s002.docx]

**Table S2 the e-value and similarities of nine test genes with their orthologous in other insects. We used the mRNA sequences to BlastX against the nr database and obtained the following results.**

| Gene | GenBank ID | Blast results | | | | |
| --- | --- | --- | --- | --- | --- | --- |
|  |  | GenBank ID | Species | Description | E-Value | Max ident |
| arf1 | JQ653045 | BAM19534.1 | *Papilio xuthus* | ADP ribosylation factor | 1e-122 | 99% |
|  |  | XP_003396009.1 | *Bombus impatiens* | PREDICTED: ADP-ribosylation factor-like protein 1-like | 3e-114 | 91% |
|  |  | XP_001120141.1 | *Apis mellifera* | PREDICTED: ADP-ribosylation factor-like protein 1-like isoform 1 | 1e-113 | 91% |
|  |  | XP_320779.3 | *Anopheles gambiae str. PEST* | AGAP011730-PA | 3e-113 | 91% |
|  |  | EGI68757.1 | *Acromyrmex echinatior* | ADP-ribosylation factor-like protein 1 | 7e-113 | 91% |
| arf2 | JF915770 | NP_001091755.1 | *Bombyx mori* | ADP-ribosylation factor | 8e-124 | 100% |
|  |  | EHJ64178.1 | *Danaus plexippus* | ADP-ribosylation factor | 3e-123 | 99% |
|  |  | AFO83996.1 | *Antheraea yamamai* | ADP-ribosylation factor, partial | 5e-123 | 100% |
|  |  | NP_476955.1 | *Drosophila melanogaster* | ADP-ribosylation factor 79F | 8e-123 | 99% |
|  |  | ADE42873.1 | *Marsupenaeus japonicus* | ADP-ribosylation factor 1 | 9e-123 | 99% |
| tubulin1 | JQ653042 | EHJ77543.1 | *Danaus plexippus* | hypothetical protein KGM_20194 | 0 | 96% |
|  |  | XP_394981.4 | *Apis mellifera* | PREDICTED: tubulin gamma-1 chain isoform 1 | 0 | 82% |
|  |  | EFN75928.1 | *Harpegnathos saltator* | Tubulin gamma-1 chain | 0 | 82% |
|  |  | XP_001601917.1 | *Nasonia vitripennis* | PREDICTED: tubulin gamma-1 chain-like | 0 | 82% |
|  |  | XP_003695179.1 | *Apis florea* | PREDICTED: LOW QUALITY PROTEIN: tubulin gamma-1 chain-like | 0 | 82% |
| tubulin2 | JQ653043 | BAM17974.1 | *Papilio xuthus* | beta-tubulin | 4e-166 | 98% |
|  |  | EHJ73579.1 | *Danaus plexippus* | beta-tubulin | 5e-166 | 98% |
|  |  | NP_001036888.1 | *Bombyx mori* | beta-tubulin | 3e-165 | 98% |
|  |  | XP_314483.4 | *Anopheles gambiae str. PEST* | AGAP010510-PA | 9e-159 | 93% |
|  |  | XP_967348.1 | *Tribolium castaneum* | PREDICTED: similar to AGAP010510-PA | 3e-158 | 92% |
| chitinase1 | JQ653040 | EHJ65741.1 | *Danaus plexippus* | chitinase-related protein 1 | 0 | 68% |
|  |  | EFA10488.1.1 | *Tribolium castaneum* | hypothetical protein TcasGA2_TC012734 | 0 | 63% |
|  |  | CAD31740.4 | *Tenebrio molitor* | chitinase | 0 | 66% |
|  |  | XP_001655973.1 | *Aedes aegypti* | brain chitinase and chia | 0 | 64% |
|  |  | EFZ20273.1 | *Solenopsis invicta* | hypothetical protein SINV_16002 | 0 | 62% |
| chitinase7 | JQ653039 | AFI55112.1 | *Plutella xylostella* | chitinase | 0 | 91% |
|  |  | XP_308858.4 | *Anopheles gambiae str. PEST* | AGAP006898-PA | 0 | 77% |
|  |  | [NP_001036035.1](http://www.ncbi.nlm.nih.gov/protein/110431374?report=genbank&log$=prottop&blast_rank=6&RID=KDC5UNHB01R) | *Tribolium castaneum* | chitinase 7 precursor | 0 | 81% |
|  |  | XP_002425481.1 | *Pediculus humanus corporis.* | conserved hypothetical protein | 0 | 79% |
|  |  | XP_001950380.1 | *Acyrthosiphon pisum* | hypothetical protein LOC100165452 | 0 | 76% |
| PGCP | JQ653044 | EHJ64655.1 | *Danaus plexippus* | putative plasma glutamate carboxypeptidase | 2e-151 | 60% |
|  |  | AEE61861.1 | *Dendroctonus ponderosae* | unknown | 6e-127 | 52% |
|  |  | XP_001601839.2 | *Nasonia vitripennis* | PREDICTED: plasma glutamate carboxypeptidase-like | 5e-125 | 52% |
|  |  | XP_003696161.1 | *Apis florea* | PREDICTED: plasma glutamate carboxypeptidase-like | 2e-123 | 52% |
|  |  | XP_971371.1 | *Tribolium castaneum* | PREDICTED: similar to predicted protein | 2e-121 | 52% |
| helicase | JF915771 | EHJ70266.1 | *Danaus plexippus* | hypothetical protein KGM_18078 | 0 | 88% |
|  |  | XP_396195.3 | *Apis mellifera* | PREDICTED: chromatin-remodeling complex ATPase chain Iswi isoform 1 | 0 | 81% |
|  |  | XP_003695213.1 | *Apis florea* | PREDICTED: chromatin-remodeling complex ATPase chain Iswi-like | 0 | 81% |
|  |  | [XP_001648130.1](http://www.ncbi.nlm.nih.gov/protein/157103787?report=genbank&log$=prottop&blast_rank=11&RID=KD6VC0SS016) | *Aedes aegypti* | helicase | 0 | 82% |
|  |  | [XP_972116.1](http://www.ncbi.nlm.nih.gov/protein/91081375?report=genbank&log$=prottop&blast_rank=16&RID=KD6VC0SS016) | *Tribolium castaneum* | PREDICTED: similar to helicase | 0 | 87% |
| ATPase | JQ653046 | EHJ74490.1 | *Danaus plexippus* | hypothetical protein KGM_18978 | 0 | 92% |
|  |  | EEZ97214.1 | *Tribolium castaneum* | hypothetical protein TcasGA2_TC011007 | 0 | 80% |
|  |  | EGI65273.1 | *Acromyrmex echinatior* | Putative phospholipid-transporting ATPase ID | 0 | 76% |
|  |  | XP_003692301.1 | *Apis florea* | PREDICTED: probable phospholipid-transporting ATPase ID-like | 0 | 74% |
|  |  | XP_396773.3 | *Apis mellifera* | PREDICTED: probable phospholipid-transporting ATPase ID-like isoform 1 | 0 | 74% |
| G3PDH | scaffold925 | [BAD38675.1](http://www.ncbi.nlm.nih.gov/protein/51555848?report=genbank&log$=prottop&blast_rank=1&RID=NNSRWSXD01R) | *Bombyx mori* | glycerol-3-phosphate dehydrogenase-2 | 2e-143 | 91% |
|  |  | [XP_975007.1](http://www.ncbi.nlm.nih.gov/protein/91076880?report=genbank&log$=prottop&blast_rank=3&RID=NNSRWSXD01R) | *Tribolium castaneum* | PREDICTED: similar to glycerol-3-phosphate dehydrogenase | 4e-126 | 80% |
|  |  | [XP_003397689.1](http://www.ncbi.nlm.nih.gov/protein/340718469?report=genbank&log$=prottop&blast_rank=5&RID=NNSRWSXD01R) | *Bombus terrestris* | PREDICTED: glycerol-3-phosphate dehydrogenase [NAD+], cytoplasmic-like | 3e-118 | 78% |
|  |  | [XP_003486248.1](http://www.ncbi.nlm.nih.gov/protein/350401748?report=genbank&log$=prottop&blast_rank=6&RID=NNSRWSXD01R) | *Bombus impatiens* | PREDICTED: glycerol-3-phosphate dehydrogenase [NAD+], cytoplasmic-like | 3e-118 | 78% |
|  |  | [XP_001653595.1](http://www.ncbi.nlm.nih.gov/protein/157120299?report=genbank&log$=prottop&blast_rank=7&RID=NNSRWSXD01R) | *Aedes aegypti* | glycerol-3-phosphate dehydrogenase | 1e-117 | 77% |
| E2F | C6515767 | [NP_001040298.1](http://www.ncbi.nlm.nih.gov/protein/114051451?report=genbank&log$=prottop&blast_rank=1&RID=NNSV8Y6U015) | *Bombyx mori* | E2F transcription factor 4-like protein | 6e-48 | 82% |
|  |  | [EHJ67877.1](http://www.ncbi.nlm.nih.gov/protein/357612247?report=genbank&log$=prottop&blast_rank=2&RID=NNSV8Y6U015) | *Danaus plexippus* | E2F transcription factor 4-like protein | 1e-45 | 86% |
|  |  | [XP_001627712.1](http://www.ncbi.nlm.nih.gov/protein/156368461?report=genbank&log$=prottop&blast_rank=3&RID=NNSV8Y6U015) | *Nematostella vectensis* | predicted protein | 2e-44 | 73% |
|  |  | [EGI62724.1](http://www.ncbi.nlm.nih.gov/protein/332022416?report=genbank&log$=prottop&blast_rank=4&RID=NNSV8Y6U015) | *Acromyrmex echinatior* | Transcription factor E2F5 | 3e-43 | 73% |
|  |  | [EFN84044.1](http://www.ncbi.nlm.nih.gov/protein/307205886?report=genbank&log$=prottop&blast_rank=5&RID=NNSV8Y6U015) | *Harpegnathos saltator* | Transcription factor E2F4 | 2e-42 | 72% |
